# A Novel Proposal of Salivary Lymphocyte Detection and Phenotyping in Patients Affected by Sjogren’s Syndrome

**DOI:** 10.3390/jcm9020521

**Published:** 2020-02-14

**Authors:** Elena Selifanova, Tatjana Beketova, Gianrico Spagnuolo, Stefania Leuci, Anna Turkina

**Affiliations:** 1 Department of Therapeutic Dentistry, I.M. Sechenov First Moscow State Medical University (Sechenov University), 119991 Moscow, Russia; selifana@mail.ru (E.S.); anna@turkin.su (A.T.); 2 V.A. Nasonova Research Institute of Rheumatology, 119991 Moscow, Russia; tvbek22@rambler.ru; 3 Department of Neuroscience, Reproductive and Odontostomatological Sciences, Oral Medicine Unit, Federico II University of Naples, 80131 Naples, Italy; ste.leuci@gmail.com

**Keywords:** salivary glands, saliva, lymphocytes, Sjogren’s syndrome, chronic sialadenitis

## Abstract

A preliminary evaluation of the parotid secretion cellular composition in patients with Sjogren’s Syndrome (SS) and a diagnostic accuracy assessment of salivary lymphocyte detection and immunophenotyping in Sjogren’s Syndrome diagnosis and prognosis were performed. The study included 40 consecutive patients, aged 19–60 years, with parenchymal sialadenitis associated with Sjogren’s Syndrome, and 20 healthy donors. The exclusion criteria were exacerbation of sialadenitis, chronic infections, malignant neoplasms, and lymphoproliferative diseases. The standard diagnostic tests were minor salivary gland biopsy and parotid sialography. Immunophenotyping of parotid secretion lymphocytes was performed by multicolor flow cytometry. Lymphocytes were detectable in parotid secretion of patients affected by Sjogren’s Syndrome, both primary (pSS) and secondary (sSS) form, but not in that from healthy donors. Sensitivity, specificity, positive, and negative predictive values of lymphocytes detection in parotid saliva were 77.5%, 100%, 100%, and 69%, respectively. The mean numbers of the total T-cell population, T-helper cells, and T-cytotoxic cells were 71.7%, 41.6%, and 53%, respectively. The immunophenotype of lymphocytes obtained by patients’ parotid flow resembles the immunophenotypes of glandular biopsies currently known. Our preliminary data suggest the use of saliva as an alternative and non-invasive method for evaluating the prognosis of Sjogren’s Syndrome.

## 1. Introduction

Xerostomia is one of the main symptoms of Sjogren’s syndrome and can suggest clinical diagnosis of the disease [1,2].

Salivary glands (SGs) are one of the main target organs in Sjögren’s syndrome (SS), both in primary (pSS) and secondary (sSS) Sjogren’s syndrome, and the typical SG lesion is autoimmune sialadenitis [3]. Chronic focal periductal lymphocytic sialadenitis is a peculiar morphological pattern of SS which is included in all of the classification criteria of the disease, even if a lack of a gold standard for diagnosis still exists [4].

SS is considered as a multi-organ autoimmune disease, characterized by focal lymphoplasmacytic epithelial gland infiltration and polyclonal B-cell activation, with the formation of a large number of antibodies, polyclonal, and monoclonal immunoglobulins, predominantly of IgG/M class [5,6]. Thus, characteristic features are sicca symptoms, including dry eyes and a dry mouth.

In the salivary and lacrimal glands, focal lymphocytic infiltration is associated with an imbalance of cellular immune responses and chronic inflammatory reaction that can lead to lymphoid neogenesis and the formation of a tertiary lymphoid tissue. Despite the different proposed diagnostic criteria, biopsy of SGs is still a key point to detect and confirm the disease. As a semi-quantitative technique, it provides high disease specificity, wide availability, and prediction of non-Hodgkin’s lymphoma development with the presence of lymphoid germinal centers in the glands [7]. On the contrary, labial minor salivary biopsy it is an invasive procedure with the reported risk of adverse reactions such as paresthesia, wound and infected mucosa, bleeding, and retained suture mucosa [8,9]. As the composition of saliva undergoes changes in patients with SS, the study of saliva biomarkers is a promising noninvasive method for analysis of pathological processes in the disease, as well as in a wide range of other conditions [10]. Saliva collection is a simple, convenient, painless, and safe procedure, where the presence of some substances (i.e., antibodies, hormones, drugs) can correlate with their concentration in the bloodstream [11]. Advances in proteomics in recent years have prompted numerous studies of the components of saliva [12]; however, only a few works have been devoted to the study of the cellular composition of saliva [13,14,15]. Detection of lymphocyte phenotypes is currently applied to the study of primary immunodeficiencies [16,17]; otherwise, phenotyping of lymphocytes may be interested in different autoimmune disorders—in particular, SS phenotyping of lymphocytes obtained by salivary flow may be useful for diagnosis and be significant as a prognostic factor.

The purpose of this study was a preliminary evaluation of the parotid secretion cellular composition in patients with Sjogren’s Syndrome and a diagnostic accuracy assessment of salivary lymphocyte immunophenotyping in Sjogren’s Syndrome prognosis.

The study hypothesis was that salivary lymphocyte immunophenotyping is a new non-invasive diagnostic method which can be used to define inflammation or lymphoproliferation in the salivary glands.

## 2. Experimental Section

### 2.1. Study Population

The prospective study included 40 consecutive patients with primary or secondary SS, followed at the V.A. Nasonova Research Institute of Rheumatology (NRIR, Moscow, Russia). The protocol was approved by the Local Ethical Committee of the V.A. Nasonova Research Institute for Rheumatology (Protocol No10, 14.04.2016). The study was performed in the period from January 2017 to January 2018. The inclusion criteria were the following: aged 19–60 years, and parenchymal sialadenitis associated with pSS or sSS. The exclusion criteria were acute stage of salivary gland disease, presence of chronic infections (hepatitis B and C viruses, HIV, tuberculosis), malignant neoplasms, lymphoproliferative diseases, past head and neck radiation treatment, sarcoidosis, graft versus host disease, and current use of anticholinergic drugs.

The diagnosis both of pSS and sSS was confirmed according to the 2016 American College of Rheumatology (ACR)/European League Against Rheumatism (EULAR) Classification Criteria for Primary Sjögren’s Syndrome, which are also applicable for secondary Sjogren’s Syndrome [1]. These criteria were developed and validated by international working group. They are based on 2002 American-European Consensus Group (AECG) criteria [18] and 2012 ACR criteria [8]. The new criteria use a weighted some of objective symptoms, and are applicable for early detection of SS [2]. Each patient was examined by a rheumatologist, an ophthalmologist, and a dentist using a blood test, Schirmer’s test, vital dye staining of the eye surface, sialometry, and the morphological analysis of the biopsy specimen of the small salivary gland taken from the mucosa of the lower lip. We considered as diagnostic the following criteria: positive Anti-SSA (Ro) in the blood test, ocular staining score ≥5, abnormal Schirmer’s test (without anesthesia; ≤5 mm/5 min), minor salivary gland biopsy showing focal lymphocytic sialadenitis (focus score ≥1 per 4 mm^2^), and unstimulated whole salivary flow (≤0.1 mL/minute). To assess the presence and stage of pathological changes in parotid SG (PSG), we used Sialography with the introduction of a contrast medium (Omnipack 350) [19] and stimulated parotid sialometry with Lashley cup [19,20,21].

The diagnoses of rheumatoid arthritis, systemic scleroderma, and systemic lupus erythematosus in patients with sSS were confirmed by a rheumatologist according to 2010 ACR/EULAR Classification Criteria for Rheumatoid Arthritis [22], 2013 ACR/EULAR classification criteria for systemic sclerosis [23], and 2012 Systemic Lupus Collaborating Clinics (SLICC) for systemic lupus erythematosus [24], respectively.

The control group consisted of healthy volunteers from the staff of NRIR without signs of SGs and somatic pathology. The inclusion criteria for the control group were: female sex, aged 19–60 years, normal salivary glands (according to clinical examination and unstimulated salivary flow rate >0.1 mL/min), absence of systemic autoimmune diseases (negative Anti-SSA (Ro)), normal Shrimer’s test, and normal ocular staining score. Minor salivary gland biopsy was not provided. The exclusion criteria for the control group were the presence of any systemic infectious diseases (hepatitis B and C viruses, HIV, tuberculosis), malignant neoplasms, lymphoproliferative diseases, past head and neck radiation treatment, sarcoidosis, graft versus host disease, and current use of anticholinergic drugs.

### 2.2. Saliva Collection and Analysis

Saliva sampling was carried out in the morning on an empty stomach. The parotid Sg (PSG) secretion was obtained using the modified Lashley cup. The cannula was attached to the mucous membrane of the cheek in the mouth area of the excretory duct of PSG with stimulation of 3% ascorbic acid solution. Saliva was collected for 5 min in dry glass graduated test tubes, followed by their transportation in a thermostatic container.

The samples for the study were prepared within 2 h after the saliva sampling procedure. After a 10-fold dilution with phosphate-buffered saline (PBS), saliva samples were centrifuged at 500*g* for 5 min. After removal of the supernatant, 50 μL of PBS was added to the pellet. The analysis of lymphocyte subpopulations was carried out in the immunological laboratory of NRIR. Immunophenotyping of lymphocytes, including determination of the percentage of the total population of T-cells (CD3+), T-helpers (CD3+CD4+), T-cytotoxic cells (CD3+CD8+), natural killer cells (CD3-CD56+), and B-cells (CD3-CD19+), was performed using multicolor flow cytometry on the NAVIOS analyzer (Beckman Coulter, USA). The commercial kits of mouse monoclonal antibodies were used: CYTO-STAT tetraCHROME CD45-FITC/CD4-RD1/CD8-ECD/CD3-PC5 (Beckman Coulter, USA) and CYTO-STAT tetraCHROME CD45-FITC/CD56-RD1/CD19-ECD/CD3-PC5 (Beckman Coulter, USA). The immunoregulatory index was calculated as a ratio of T-helpers and T-cytotoxic cells (CD3+CD4+/CD3+CD8+). The value of CD4/CD8 index of 1.5–2.5 was considered as an indicator of a normal state, more than 2.5 (hyperactivity state) and less than 1.5 (immunodeficiency state).

The same samples of saliva were also analyzed by a cytologist. The presence of different cells in salivary sediment and the percentage of lymphocytes in the whole saliva were recorded.

The presence of any lymphocytes in the saliva is supposed to be a sign of inflammation in the salivary gland. Parotid secretion, in contrast to the whole saliva, is sterile and contains no cells [25].

According to the 2016 American College of Rheumatology/European League Against Rheumatism Classification Criteria for Primary and Secondary Sjögren’s Syndrome, the reference standard for salivary gland condition assessment were the following: minor salivary gland biopsy showing focal lymphocytic sialadenitis (focus score ≥1 per 4 mm^2^) and unstimulated whole salivary flow (≤0.1 mL/minute). For the assessment of parotid salivary glands, we also used parotid sialography and stimulated parotid sialometry.

Clinical information was not available to the performers of the test. However, clinical information and index test results were available to the assessors of the test and reference standards.

The diagnostic accuracy was estimated by calculation of sensitivity, specificity, positive, and negative predicative values. The results were processed using the Statistica 10 statistical software package (StatSoft, Inc. Tusla, OK, USA). The sample size was not determined before the study beginning. Sample size was limited by the time of the study. Also, there were no missing data in the sample.

## 3. Results

### 3.1. Study Population

**Figure 1:**
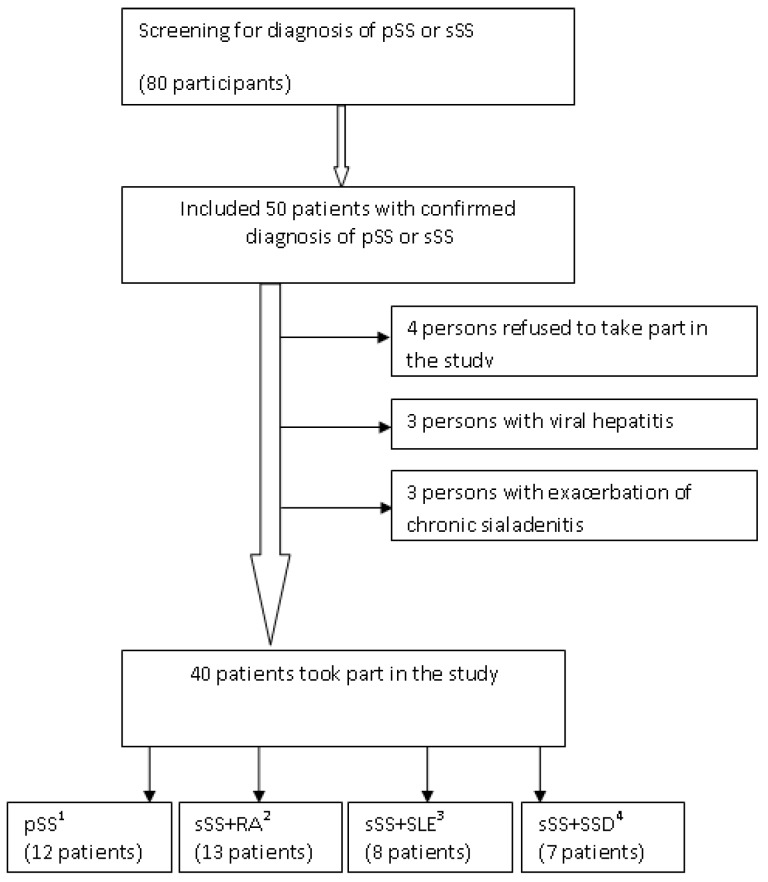
Patient flow diagram. ^1^ primary Sjogren’s syndrome; ^2^ secondary Sjogren’s syndrome, associated with rheumatoid arthritis; ^3^ secondary Sjogren’s syndrome, associated with systemic lupus erythematosus; ^4^ secondary Sjogren’s syndrome, associated with systemic scleroderma.

A total of 80 patients underwent screening examination in the V.A. Nasonova Research Institute of Rheumatology, and after the complete examination, 50 patients had a confirmed diagnosis of pSS or sSS. Ten patients were excluded (see patient flow diagram in Figure 1). Finally, the test group included 40 patients with parenchymal sialadenitis, including 12 cases of pSS and 28 cases of sSS combined with RA (13 cases), SLE (8 cases), and SSD (7 cases). The diagnosis of pSS or sSS was confirmed at least 5 years ago. The control group included 40 female patients. The mean age of the patients was 47.7 ± 12.9 in the test group and 47.6 ± 16.6 in the control group.

All of the patients with pSS and sSS received systemic therapy. The total duration of systemic therapy varied from four to seven years (one cycle every six months). The saliva was obtained during patient examination before the following treatment cycle.

Using sialography, we revealed stages of parenchymatous parotitis (Figure S1). In total, the severity of parenchymatous parotitis was the following: I, II, and III stages were defined in 12, 20, and eight patients, respectively.

In the morphological study of biopsy specimens of small SG in patients with pSS and sSS, periductal lymphohystocyte infiltrates were observed, which included 50 to 150 cells, depending on the disease stage (Figure S2).

In all of the patients affected with SS, salivary secretion rate was decreased. The lowest secretion level was measured in group with PSS. The baseline demographic and clinical characteristics of the participants are summarized in Table 1.

There were no significant time distances or any additional treatment between baseline examination and the index test. Parotid saliva used for immunophenotyping was collected at the same time when sialometry was provided.

### 3.2. The Cellular Composition of Saliva

Cytological evaluation of salivary sediment revealed lymphocytes in 80% of patients with pSS and sSS. Lymphocytes were completely absent in the control group. In addition, epithelial cells (97.5%), erythrocytes (7.5%), and granulocytes (37.5%) were detected. In healthy patients, only epithelial cells (55%) and granulocytes (1 case, 5%) were detected (Table S1). In the majority of patients with SS, lymphocytes took up to 10% of all detected cells. However, we also observed seven patients with 11–50% of lymphocytes in cellular composition of parotid saliva (Table S2). In two patients, lymphocytes took more than 50% of cells in salivary sediment (one patient with pSS and one patient with sSS associated with lupus erythematosus).

Immunophenotyping of lymphocytes showed that the average incidence of T-cells (CD3+) in the general population was 71.7%, T-helpers (CD3 + CD4+) 41.6%, and T-cytotoxic cells (CD3 + CD8+) 53% (Table S3, Figure S3, Figure S4).

In pSS, by contrast to sSS, we revealed a predominance of the general population of CD3+ T-cells (90% and 64.5–66.8%, respectively), mainly due to an increase in the number of CD8+ cytotoxic cells (64% and 51.4–47.8%, respectively). The ratio of the relative number of subpopulations of T-helpers (CD4+) and cytotoxic T-lymphocytes (CD8+) varied in the range of 0.68–0.94 (average 0.87). The lowest ratio of CD4+/CD8+ (0.68) was observed in patients with sSS. The natural killer cells (CD56+) and B-lymphocytes (CD19+) were present in a relatively small amount only in patients with sSS (9% and 9%, respectively) and sSS/RA (8.7% and 2.2%, respectively). All of the patients with B-lympocytes had Grade 3 parotitis according to parotid sialography.

The preliminary calculation of the diagnostic accuracy of salivary lymphocytes detection showed the following results. Sensitivity level was 77.5%, specificity was 100%, positive predictive value (PPV) was 100%, and negative predictive value (NPV) was 69% (Table 2).

The index test provided no indeterminate results, because the presence of any lymphocytes was supposed to be a positive result. The reference standard tests were assessed in complex, so indeterminate results were also absent.

No adverse effects of the test were observed.

## 4. Discussion

SS is a relatively common disease. Among the autoimmune rheumatic diseases, the disease is possibly the second most common, only surpassed by rheumatoid arthritis [26]. The SS affects both the minor and major salivary glands, causing autoimmune sialadenitis [3].

Saliva reflects the state of the oral cavity, the salivary glands, and the whole body. Pathological changes of SG in patients with SS have a certain impact on the composition of the saliva [10]. It should be noted that there are some differences in composition of whole and glandular saliva. Whole saliva is composed of secretions of major and minor SG, gingival crevicular fluid, food and debris [27], and, in contrast to glandular saliva, contains a lot of cells obtained from oral mucosa and gingival sulcus [28]. The pure parotid saliva, which was used for the present study, is less affected by local processes in the oral cavity than the whole saliva. The pure parotid saliva does not contact periodontium, cervical crevicular fluid, or oral mucosa, and has a different proteomic profile compared to the whole saliva [27]. The impact of topical oral processes on the parotid saliva composition was evaluated in several studies. In the literature, there are reports about immunological and biochemical changes of the parotid and whole saliva in patients with periodontal disease [29,30], but it was also mentioned that periodontal treatment does not affect parotid saliva composition [31]. Whole saliva cellular compound strongly depends on the topical inflammation, such as gingivitis [15]. Whole saliva cell immunophenotyping revealed that patients with CP had a higher frequency of total leukocytes, B-cells, NK cells, and CD4(+) T-cells than individuals without oral pathologies [14]. The lymphocytes in whole saliva are probably obtained from junctional epithelium [32], gingival crevicular fluid [33], and oral mucosa [34], which contain lymphocytes as components of host defense. However, we did not find in the available literature any studies according to lymphocytes in pure parotid saliva in healthy and periodontal patients. Thus, we analyzed pure parotid saliva because it better corresponds to the condition of parotid SG.

In the parotid secretion, we observed a predominance of T-cells with a decrease in the ratio of CD4+/CD8+ cell subpopulations (0.87 on average), with the lowest values observed in patients with pSS (0.68). The presence of an insignificant amount of B-lymphocytes (CD19+) in the secretion of the parotid gland is observed only in patients with pSS and sSS/RA (9% and 2.2%, respectively).

The development of lymphocytic sialadenitis in SS is a multistage process that involves the formation of a small, scattered perivascular lymphoid infiltrate, the sequential development of a typical focal periductal lymphoid sialadenitis, and then a diffuse lymphocytic sialadenitis with the formation of ectopic embryonic centers, which finally leads to the destruction and replacement of the affected glandular tissue. The lymphoid infiltrates at the onset of the disease include predominantly activated CD4+ (CD45Ro+) T-cells that predominate over T-suppressors; in addition, CD8+ T-cells are invariably present [5,7,35]. The severe course is accompanied by an increase in the population of B-lymphocytes and macrophages [36], while in advanced stages of the disease, B-cells and plasma cells prevail, especially when the ectopic embryonic centers are formed. Epithelial cells of SG are activated, with impaired apoptosis and the ability to stimulate adhesion with the function of antigen-presenting cells and co-stimulation of infiltrating CD4+ T-cells. However, infiltrating CD4+ T-cells and dendritic cells can also locally produce a wide range of cytokines targeting B-cells, including BAFF and APRIL. Close interaction between activated epithelial cells of SG, infiltrating lymphocytes and dendritic cells leads to chronic inflammation and progression of the disease [5,7].

The results of immunophenotyping of lymphocytes of parotid secretion can be considered as an indicator of a chronic inflammatory reaction. The presence of T-cells in the parotid secretion can be partially explained by the results of the experimental study provided by Wang et al. in 2018. The authors used a mouse model where A20 was knocked out under control of the keratin 14 (KRT14) promoter. In the biopsy specimens of submandibular SG, CD3^+^ T-cells were both found in foci and dispersed through the gland. Invasion of striated ducts was also observed. Immunostaining for CD3 and B220 revealed that T-cells and occasional B-cells were observed invading striated ducts. It was concluded that immune activation of dysregulated epithelial cells culminating in augmented NFκB pathway activity is sufficient to predispose the salivary gland for the development of an inflammatory immune milieu [37]. Nandula et al., in 2011, in a mouse model showed that innate immunity activation can cause the initial inflammatory cell infiltration of submandibular SG followed by CD4+ T-cells [38]. In a clinical study by van Ginkel et al. [39], biopsy specimens of parotid and labial SG of patients with SS were compared. The presence of B-cells was strongly associated with lympho-epithelial lesions, and T-lymphocytes were detected in all striated ducts without hyperplasia and striated ducts with lymphoepithelial lesions. B-lymphocytes in striated ducts with lymphoepithelial lesions (LELs) were mostly concentrated in the areas where the epithelium was proliferating, whereas T-lymphocytes were scattered through the whole ductal epithelium [39]. It was previously reported that the biopsy specimens of the parotid and small SGs provide similar histological features, sensitivity, and specificity [40]. Nevertheless, van Ginkel et al. mentioned that numbers of B-lymphocytes, T-lymphocytes, and B/T ratios within LELs were significantly higher in the parotid gland than in the labial gland [39]. This finding corresponds to the opinion of Marx et al., who showed that parotid biopsy identified pSS in an earlier stage, and with more evident histopathology than labial SG biopsy [41]. In our study, we did not find a correlation between lymphocytes in parotid saliva and degree of pathological changes in the minor salivary gland.

The role of T-cells in SS remains questionable because we do not know yet if this type of infiltrate is specific, if this expansion occurs within the SG or in periphery with subsequent migration, if there is a specific antigen driving T-cell expansion, and why some T-cells are involved in parenchyma destruction and/or are the primum movens of B-cell activation. We can also hypothesize that the inflammatory process in salivary glands continues despite the treatment, and T-cells which are located mainly around the striated ducts can penetrate into the duct lumen in the result of the destructive process in SG. However, the predictive value of cytological biomarkers for the development and course of Sjögren’s syndrome remains questionable and needs further investigation [42].

The decrease of CD4/CD8 ratio in the blood due to the increased level of CD8+ is considered a marker for a number of tumors, that is, of particular significance for pSS which is associated with the highest risk of developing lymphoproliferative diseases compared to sSS, RA, SLE, or other systemic connective tissue diseases [43].

An important factor which can affect the lymphocyte profile of the salivary glands and saliva is systemic therapy, which usually includes cytostatics and immunobiologics (Anti-B-cell agents, Anti-TNF, and others). The use of immunobiologics in SS patients is the less studied area. We suppose that parotid saliva immunophenotyping could be used for monitoring of patients treated with immunobiologics [44].

The determination of the disease activity and prognosis in patients affected with SS is a current issue in rheumatology. In 2009–2011, EULAR Sjogren’s syndrome disease activity index and EULAR Sjogren’s Syndrome Patient Reported Index (ESSPRI) were developed. According to these indices, the oral component of SS can be assessed only by two criteria: glandular swelling and subjective oral dryness [45,46]. Thus, some objective criteria were needed to assess SG condition and treatment effect, especially in clinical trials [47,48]. PSG biopsy is supposed to be a relevant method because of the possibility of multiple biopsies, which can demonstrate the changes in lymphoid infiltrate [49,50]. Several studies also reported the importance of parotid biopsy for lymphoma diagnosis [51,52]. As the parotid secretion represents the condition of PSG itself, immunophenotyping of salivary lymphocytes could be an alternative to parotid biopsy in the monitoring of patients affected by SS. The total number and types of lymphocytes in the parotid secretion could be used to evaluate the treatment effect. The appearance of B-cells in parotid secretion in patients receiving cytostatic therapy could be an indication for PSG biopsy.

In our study we used PSG sialography to define the parotitis stage. B-cells were detected in the secretion of patients with Grade 3 parotitis only when the distraction of ducts and contrast penetration into parenchyma were revealed. Another non-invasive method for PSG assessment is ultrasonography which was supposed to be useful for early diagnosis of SS and evaluating of treatment effects [53,54]. Since the ultrasonographic studies demonstrated the correlation with minor SG and PSG biopsies [55] it would be interesting to evaluate possible correlations between salivary lymphocytes detection and parotid ultrasonography.

Overall, the profile of pathological changes in parotid secretion revealed in this work by immunophenotyping of lymphocytes corresponds to the previously published results of immunohistochemical studies of biopsy specimens. This result suggests a significant potential of saliva analysis as a method of choice for diagnosing and evaluating the prognosis of patients with SS. Our results show that there is a strong correlation among minor salivary gland histological phenotype, the prevalence of B-cells, and the very high level of anti-Ro autoantibodies in blood, mainly in Stage 3 parotitis patients. The saliva lymphocyte phenotyping could be, for this reason, a very useful test in the evaluation of the disease progression, allowing clinicians to better orient the treatment.

The main limitations of the present study were the small sample size and difficulties with the interpretations of the test results. We can suppose that the presence of the T-lymphocytes in the parotid saliva can be a sign of the inflammatory process. B-lymphocytes may be a possible sign of lymphoma. The following issue should be also noted. In the present study we evaluated CD19+ B-cells which were previously described in PSG biopsies [56], while in the parotid gland infiltrate CD19 negative and CD138 positive plasma cells are present which play a significant role in pathological process [57]. In future other subpopulations of B-cells and plasma cells should be evaluated. Further investigations in a larger cohort are needed to assess correlations between parotid saliva and parotid biopsy, parotid saliva and minor salivary gland biopsy, and parotid saliva and blood.

## 5. Conclusions

The following important conclusions can be drawn on the basis of the results obtained in this work. In parotid saliva of patients with pSS and sSS, T-cells were detected, which were completely absent in the parotid secretion of healthy patients from the control group. In patients with pSS, T-cells in parotid secretion were detected significantly more often than in patients with sSS (90% and 65.4%, respectively), which indicates a more intensive inflammatory process even in patients treated with cytostatics. Cytotoxic T-cells in all groups prevailed over T-helper cells, and a deviation from this dependence indicates a decreased immune response and the possibility of chronic infection. The results of immunophenotyping of parotid saliva lymphocytes require further study. In general, the results of this work demonstrate that saliva analysis using the immunophenotyping technique has a high diagnostic value for a comprehensive examination of patients with SD and SS.

## Figures and Tables

**Table 1:** Main characteristics of participants.

	Groups of Patients				
	pSS ^1^	sSS + RA ^2^	sSS + SLE ^3^	sSS + SSD ^4^	Control Group
No of Patients	12	13	8	7	20
Mean Age, Years	52.1 ± 13.7	46.1 ± 13.7	45.0 ± 6.5	46.3 ± 14.3	47.7 ± 12.8
Systemic Therapy	Rituximab 500 mg at 6-month intervals	Methotrexate 10–20 mg weekly, Methylprednisolone 4 mg daily	Plaquenil 400 mg daily Methylprednisolone 4–8 mg daily	Cyclophosphamide 600–800 mg every month Methylprednisolone mg daily	None
Sialographic Stages, Abs (%)					
I	1 (8.3%)	7 (53.8%)	2 (25%)	2 (28.6%)	
II	7 (58.3%)	5 (38.5%)	7 (62.5%)	3 (42.8%)	−
III	4 (33.3%)	1 (7.7%)	1 (12.5%)	2 (28.6%)	
IV	0	0	0	0	
Stimulated Parotid Sialometry, ml/5 min	1.6 ± 0.2	2.6 ± 0.3	2.3 ± 0.2	2.4 ± 0.2	3.5 ± 0.3

^1^ Primary Sjogren’s syndrome; ^2^ secondary Sjogren’s syndrome, associated with rheumatoid arthritis; ^3^ secondary Sjogren’s syndrome, associated with systemic lupus erythematosus; ^4^ secondary Sjogren’s syndrome, associated with systemic scleroderma.

**Table 2:** Diagnostic accuracy of the salivary lymphocytes detection.

	Sjogren’s Syndrome	Control Group	Total
Positive Test	31	0	31
Negative Test	9	20	29
Total	40	20	60

## Supplementary Materials

The following are available online at https://www.mdpi.com/2077-0383/9/2/521/s1: Figure S1: Parenchymatous parotitis X-rays, Figure S2: Labial salivary gland biopsy (hematoxylin-eosin × 200), Figure S3: Representative set of scatter-plots of a patient with SS, Figure S4: The relative number of subpopulations of T-helpers (CD4+) and cytotoxic T-lymphocytes (CD8+) in the studied groups of patients. Table S1: Cytological analysis of salivary sediment in studied patients, Table S2: Lymphocytes percentage in the cellular composition of salivary sediment in studied patients; Table S3: Immunogram of parotid saliva of the studied patients.

## References

[1] Shiboski S.C., Seror R., Criswell L.A., Labetoulle M., Lietman T.M., Rasmussen A., Mariette X. (2017). 2016 American College of Rheumatology/European League Against Rheumatism Classification Criteria for Primary Sjögren’s Syndrome: A Consensus and Data-Driven Methodology Involving Three International Patient Cohorts. Arthritis Rheumatol..

[2] Franceschini F., Cavazzana I., Andreoli L., Tincani A. (2017). The 2016 classification criteria for primary Sjogren’s syndrome: what’s new?. BMC Med..

[3] Kessler A.T., Bhatt A.A. (2018). Review of the Major and Minor Salivary Glands, Part 1: Anatomy, Infectious, and Inflammatory Processes. J. Clin. Imaging Sci..

[4] Fisher B.A., Jonsson R., Daniels T., Bombardieri M., Brown R.M., Morgan P., Barone F. (2017). Standardisation of labial salivary gland histopathology in clinical trials in primary Sjögren’s syndrome. Ann. Rheum. Dis..

[5] Adamson T.C., Fox R.I., Frisman D.M., Howell F.V. (1983). Immunohistologic analysis of lymphoid infiltrates in primary Sjogren’s syndrome using monoclonal antibodies. J. Immunol..

[6] Chen X., Wu H., Wei W. (2018). Advances in the diagnosis and treatment of Sjogren’s syndrome. Clin. Rheumatol..

[7] Christodoulou M.I., Kapsogeorgou E.K., Moutsopoulos H.M. (2010). Characteristics of the minor salivary gland infiltrates in Sjogren’s syndrome. J. Autoimmun..

[8] Shiboski S.C., Shiboski C.H., Criswell L., Baer A., Challacombe S., Lanfranchi H., Daniels T. (2012). American College of Rheumatology classification criteria for Sjögren’s syndrome: A data-driven, expert consensus approach in the Sjögren’s International Collaborative Clinical Alliance cohort. Arthritis Care Res. Hoboken.

[9] Bamba R., Sweiss N.J., Langerman A.J., Taxy J.B., Blair E.A. (2009). The minor salivary gland biopsy as a diagnostic tool for Sjogren syndrome. Laryngoscope.

[10] Aqrawi L.A., Galtung H.K., Vestad B., Øvstebø R., Thiede B., Rusthen S., Jensen J.L. (2017). Identification of potential saliva and tear biomarkers in primary Sjögren’s syndrome, utilising the extraction of extracellular vesicles and proteomics analysis. Arthritis Res. Ther..

[11] Nunes L.A., Mussavira S., Bindhu O.S. (2015). Clinical and diagnostic utility of saliva as a non-invasive diagnostic fluid: A systematic review. Biochem. Med. Zagreb.

[12] Katsiougiannis S., Wong D.T. (2016). The Proteomics of Saliva in Sjögren’s Syndrome. Rheum. Dis. Clin..

[13] Kaufman E., Lamster I. (2000). Analysis of saliva for periodontal diagnosis – a review. J. Clin. Periodontol..

[14] Naiff P.F., Ferraz R., Cunha C.F., Orlandi P.P., Boechat A.L., Bertho A.L., Dos-Santos M.C. (2014). Immunophenotyping in saliva as an alternative approach for evaluation of immunopathogenesis in chronic periodontitis. J. Periodontol..

[15] Theda C., Hwang S.H., Czajko A., Loke Y.J., Leong P., Craig J.M. (2018). Quantitation of the cellular content of saliva and buccal swab samples. Sci. Rep..

[16] Hansen A., Lipsky P.E., Dorner T. (2007). B-cells in Sjogren’s syndrome: Indications for disturbed selection in ectopic lymphoid tissue. Arthritis Res. Ther..

[17] Schmid U., Helbron D., Lennert K. (1982). Development of malignant lymphoma in myoepithelial sialadenitis (Sjogren’s syndrome). Virchows Arch. Pathol. Anat. Histopathol..

[18] Vitali C., Bombardieri S., Jonsson R., Moutsopoulos H., Alexander E., Carsons S., Daniels T., Fox P., Fox R., Kassan S. (2002). Classification criteria for Sjögren’s syndrome: A revised version of the European criteria proposed by the American-European Consensus Group. Ann. Rheum. Dis..

[19] Golder W., Stiller M. (2014). Distribution pattern of Sjögren’s syndrome: A sialographical study. Z. Für Rheumatol..

[20] Aoun G., Nasseh I., Berberi A. (2016). Evaluation of the oral component of Sjögren’s syndrome: An overview. J. Int. Soc. Prev. Community Dent..

[21] Rubin P., Holt J. (1957). Secretory sialography in diseases of the major salivary glands. Am. J. Roentgenol. Radium. Ther. Nucl. Med..

[22] Kay J., Upchurch K.S. (2012). ACR/EULAR 2010 rheumatoid arthritis classification criteria. Rheumatology.

[23] Van den Hoogen F., Khanna D., Fransen J., Johnson S.R., Baron M., Tyndall A., Matucci-Cerinic M., Naden R.P., Medsger T.A., Carreira P.E. (2013). 2013 classification criteria for systemic sclerosis: An American College of Rheumatology/European League against Rheumatism collaborative initiative. Arthritis Rheum..

[24] Petri M., Orbai A.M., Alarcón G.S., Gordon C., Merrill J.T., Fortin P.R., Bruce I.N., Isenberg D., Wallace D.J., Nived O. (2012). Derivation and validation of the Systemic Lupus International Collaborating Clinics classification criteria for systemic lupus erythematosus. Arthritis Rheum..

[25] De Almeida P.V., Grégio A.M., Machado M.A., de Lima A.A., Azevedo L.R. (2008). Saliva composition and functions: A comprehensive review. J. Contemp. Dent. Pract..

[26] Fox R.I., Michelson P., Casiano C.A., Hayashi J., Stern M. (2000). Sjogren’s syndrome. Clin. Dermatol..

[27] Jasim H., Olausson P., Hedenberg-Magnusson B., Ernberg M., Ghafouri B. (2016). The proteomic profile of whole and glandular saliva in healthy pain-free subjects. Sci. Rep..

[28] Aps J., Van den Maagdenberg K., Delanghe J., Martens L. (2002). Flow cytometry as a new method to quantify the cellular content of human saliva and its relation to gingivitis. Clin. Chim. Acta.

[29] Seemann R., Hägewald S.J., Sztankay V., Drews J., Bizhang M., Kage A. (2004). Levels of parotid and submandibular/sublingual salivary immunoglobulin a in response to experimental gingivitis in humans. Clin. Oral Investig..

[30] Komine K., Kuroishi T., Ozawa A., Komine Y., Minami T., Shimauchi H., Sugawara S. (2007). Cleaved inflammatory lactoferrin peptides in parotid saliva of periodontitis patients. Mol. Immunol..

[31] Henskens Y.M., van der Weijden F.A., van den Keijbus P.A., Veerman E.C., Timmerman M.F., van der Velden U., Amerongen A.V. (1996). Effect of periodontal treatment on the protein composition of whole and parotid saliva. J. Periodontol..

[32] Nakamura M. (2018). Histological and immunological characteristics of the junctional epithelium. Jpn. Dent. Sci. Rev..

[33] Delima A.J., Van Dyke T.E. (2003). Origin and function of the cellular components in gingival crevice fluid. Periodontol. 2000.

[34] Sobkowiak M.J., Davanian H., Heymann R., Gibbs A., Emgård J., Dias J., Aleman S., Krüger-Weiner C., Moll M., Tjernlund A. (2019). Tissue-resident MAIT cell populations in human oral mucosa exhibit an activated profile and produce IL-17. Eur. J. Immunol..

[35] Theander E., Vasaitis L., Baecklund E., Nordmark G., Warfvinge G., Liedholm R., Jonsson M.V. (2011). Lymphoid organisation in labial salivary gland biopsies is a possible predictor for the development of malignant lymphoma in primary Sjögren’s syndrome. Ann. Rheum. Dis..

[36] Aqrawi L.A., Skarstein K., Øijordsbakken G., Brokstad K.A. (2013). Ro52- and Ro60-specific B cell pattern in the salivary glands of patients with primary Sjögren’s syndrome. Clin. Exp. Immunol..

[37] Wang X., Shaalan A., Liefers S., Coudenys J., Elewaut D., Proctor G.B., Bootsma H., Kroese F.G.M., Pringle S. (2018). Dysregulation of NF-kB in glandular epithelial cells results in Sjögren’s-like features. PLoS ONE.

[38] Nandula S.R., Scindia Y.M., Dey P., Bagavant H., Deshmukh U.S. (2011). Activation of innate immunity accelerates sialoadenitis in a mouse model for Sjögren’s syndrome-like disease. Oral Dis..

[39] Van Ginkel M.S., Haacke E.A., Bootsma H., Arends S., van Nimwegen J.F., Verstappen G.M., Spijkervet F.K.L., Vissink A., van der Vegt B., Kroese F.G.M. (2019). Presence of intraepithelial B-lymphocytes is associated with the formation of lymphoepithelial lesions in salivary glands of primary Sjögren’s syndrome patients. Clin. Exp. Rheumatol..

[40] Pijpe J., Kalk W.W.I., van der Wal J.E., Vissink A., Kluin P.M., Roodenburg J.L., Spijkervet F.K. (2007). Parotid gland biopsy compared with labial biopsy in the diagnosis of patients with primary Sjögren’s syndrome. Rheumatology.

[41] Marx R.E., Hartman K.S., Rethman K.V. (1988). A prospective study comparing incisional labial to incisional parotid biopsies in the detection and confirmation of sarcoidosis, Sjögren’s disease, sialosis and lymphoma. J. Rheumatol..

[42] Delli K., Villa A., Farah C.S., Celentano A., Ojeda D., Peterson D.E., Jensen S.B., Glurich I., Vissink A. (2019). World Workshop on Oral Medicine VII: Biomarkers predicting lymphoma in the salivary glands of patients with Sjögren’s syndrome-A systematic review. Oral Dis..

[43] Kauppi M., Pukkala E., Isomäki H. (1997). Elevated incidence of hematologic malignancies in patients with Sjögren’s syndrome compared with patients with rheumatoid arthritis (Finland). Cancer Causes Control.

[44] Gueiros L.A., France K., Posey R., Mays J.W., Carey B., Sollecito T.P., Setterfield J., Woo S.B., Culton D., Payne A.S. (2019). World Workshop on Oral Medicine VII: Immunobiologics for salivary gland disease in Sjögren’s syndrome: A systematic review. Oral Dis..

[45] Seror R., Ravaud P., Bowman S.J., Baron G., Tzioufas A., Theander E., Gottenberg J.E., Bootsma H., Mariette X., Vitali C. (2010). EULAR Sjögren’s Task Force. EULAR Sjogren’s syndrome disease activity index: Development of a consensus systemic disease activity index for primary Sjogren’s syndrome. Ann. Rheum. Dis..

[46] Seror R., Ravaud P., Mariette X., Bootsma H., Theander E., Hansen A., Ramos-Casals M., Dörner T., Bombardieri S., Hachulla E. (2011). EULAR Sjögren’s Task Force. EULAR Sjogren’s Syndrome Patient Reported Index (ESSPRI): Development of a consensus patient index for primary Sjogren’s syndrome. Ann. Rheum. Dis..

[47] Fisher B.A., Brown R.M., Bowman S.J., Barone F. (2015). A review of salivary gland histopathology in primary Sjo¨gren’s syndrome with a focus on its potential as a clinical trials biomarker. Ann. Rheum. Dis..

[48] Spijkervet F.K., Haacke E., Kroese F.G., Bootsma H., Vissink A. (2016). Parotid Gland Biopsy, the Alternative Way to Diagnose Sjögren Syndrome. Rheum. Dis. Clin..

[49] Pijpe J., Meijer J.M., Bootsma H., van der Wal J.E., Spijkervet F.K., Kallenberg C.G., Vissink A., Ihrler S. (2009). Clinical and histologic evidence of salivary gland restoration supports the efficacy of rituximab treatment in Sjogren’s syndrome. Arthritis Rheum..

[50] Delli K., Haacke E.A., Kroese F.G., Pollard R.P., Ihrler S., van der Vegt B., Vissink A., Bootsma H., Spijkervet F.K. (2016). Towards personalised treatment in primary Sjögren’s syndrome: Baseline parotid histopathology predicts responsiveness to rituximab treatment. Ann. Rheum Dis.

[51] Pollard R.P., Pijpe J., Bootsma H., Spijkervet F.K., Kluin P.M., Roodenburg J.L., Kallenberg C.G., Vissink A., van Imhoff G.W. (2011). Treatment of mucosa-associated lymphoid tissue lymphoma in Sjogren’s syndrome: A retrospective clinical study. J. Rheumatol..

[52] Haacke E.A., Bootsma H., Spijkervet F.K.L., Visser A., Vissink A., Kluin P.M., Kroese F.G.M. (2017). FcRL4^+^ B-cells in salivary glands of primary Sjögren’s syndrome patients. J. Autoimmun..

[53] Hammenfors D.S., Causevic H., Assmus J., Brun J.G., Jonsson R., Jonsson M.V. (2019). Assessment of major salivary gland ultrasonography in Sjögren’s syndrome. A comparison between bedside and post-examination evaluations. Clin. Exp. Rheumatol..

[54] Jonsson M.V., Baldini C. (2016). Major salivary gland ultrasonography in the diagnosis of Sjögren’s syndrome: A place in the diagnostic criteria?. Rheum. Dis. Clin..

[55] Jonsson R., Brokstad K.A., Jonsson M.V., Delaleu N., Skarstein K. (2018). Current concepts on Sjögren’s syndrome - classification criteria and biomarkers. Eur. J. Oral Sci..

[56] Hamza N., Bos N.A., Kallenberg C.G.M. (2012). B-cell populations and sub-populations in Sjögren’s syndrome. La Presse Médicale.

[57] Szyszko E.A., Brokstad K.A., Oijordsbakken G., Jonsson M.V., Jonsson R., Skarstein K. (2011). Salivary glands of primary Sjögren’s syndrome patients express factors vital for plasma cell survival. Arthritis Res. Ther..

