# Supplementary material for: A Novel Proposal of Salivary Lymphocyte Detection and Phenotyping in Patients Affected by Sjogren’s Syndrome
Source: J Clin Med. 2020 Feb 14;9(2):521. doi: 10.3390/jcm9020521 (PMC7074317; doi:10.3390/jcm9020521)
Supplement: Supplementary file 1 [file jcm-09-00521-s001.pdf]

Supplementary Materials: The following are available online at www.mdpi.com/xxx/s1, Figure S1: Parenchymatous parotitis X-rays, Figure S2: Labial salivary gland biopsy (hematoxylin-eosin × 200), Figure S3: Representative set of scatter-plots of a patient with SS, Figure S4: The relative number of subpopulations of T-helpers (CD4+) and cytotoxic T-lymphocytes (CD8+) in the studied groups of patients. Table S1: Cytological analysis of salivary sediment in studied patients, Table S2: Lymphocytes percentage in the cellular composition of salivary sediment in studied patients. Table S3: Immunogram of parotid saliva of the studied patients.

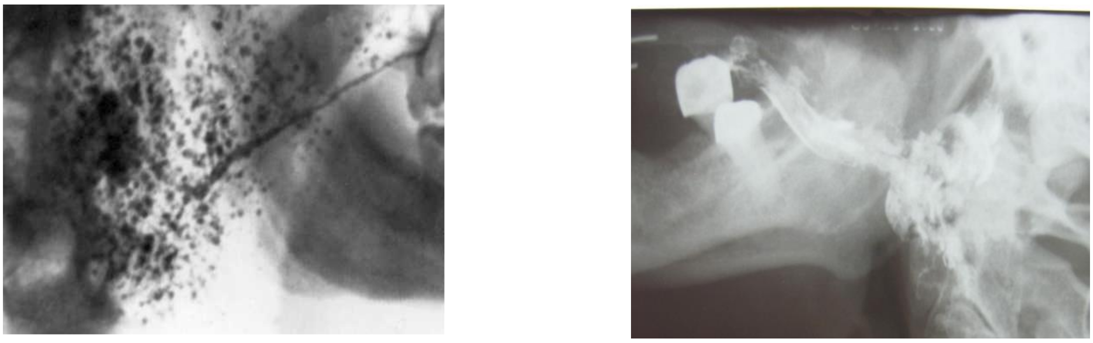

(**a**) (**b**)

Figure S1: Parenchymatous parotitis X-rays: (**a**) advanced stage, (**b**) late stage.

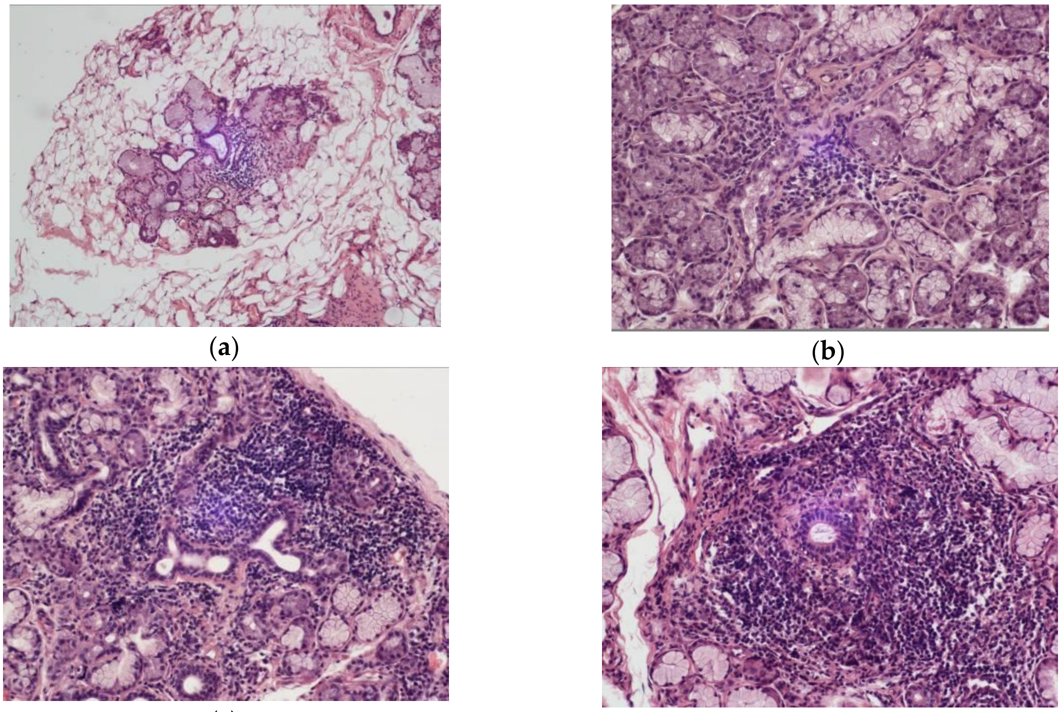

(**a**) (**b**) (**c**) (**d**)

Figure. S2: Labial salivary gland biopsy (hematoxylin-eosin × 200)**:** (**a**) enlarged intercalated ducts, focal lymphocytic infiltration, slight sialadenitis, lipid inclusions; (**b**) moderate sialadenitis, focal lymphocytic infiltration (up to 70 cells in focus); (**c**) severe sialadenitis, focal lymphocytic infiltration (up to 200 cells in focus); (**d**) severe sialadenitis, focal lymphocytic infiltration (up to 200 cells in foci, sporadic plasma cells), lymphocytic infiltration around the intercalated duct.

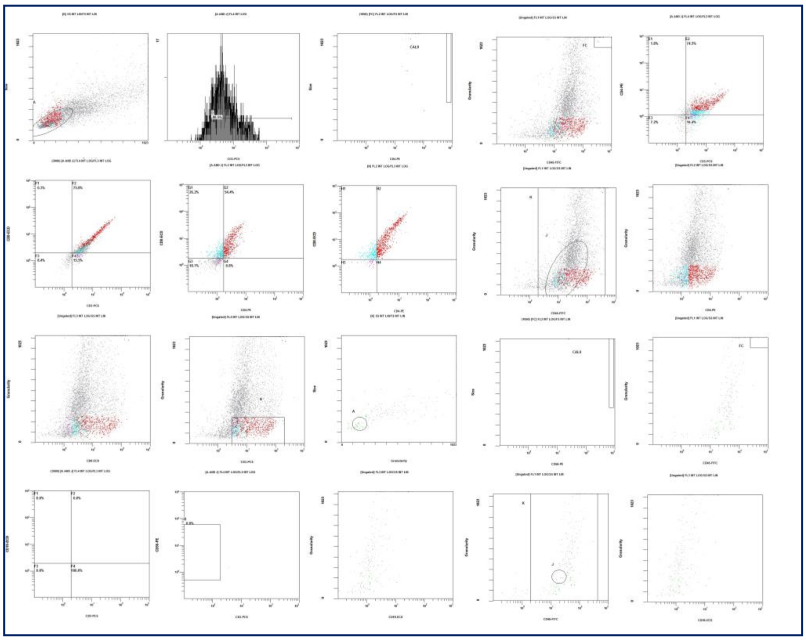

Figure S3: Representative set of scatter-plots of a patient with SS.

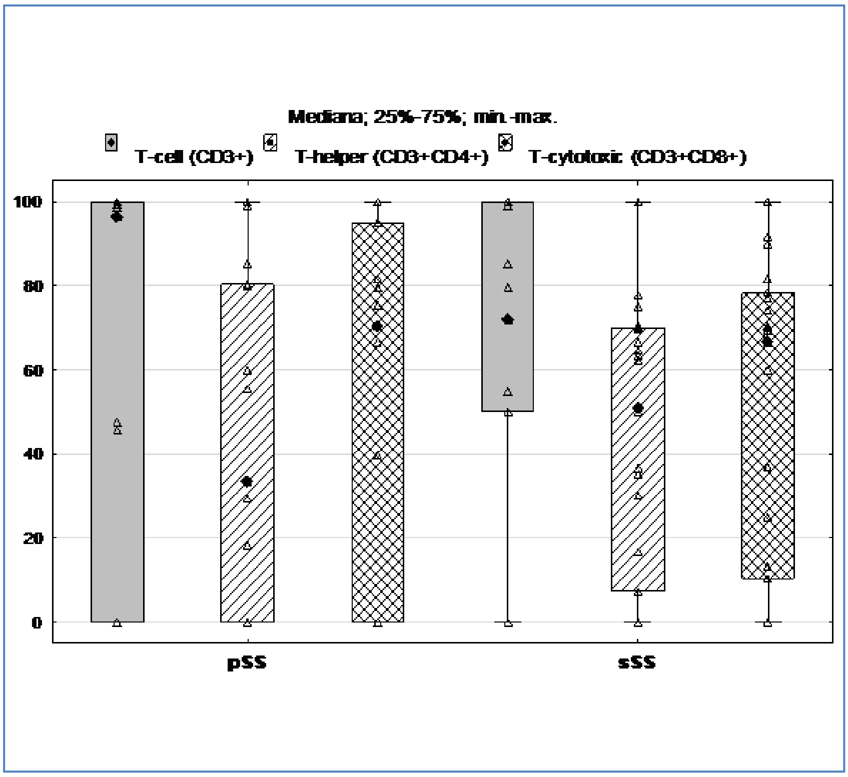

Figure S4: The relative number of subpopulations of T-helpers (CD4+) and cytotoxic T-lymphocytes (CD8+) in the studied groups of patients. pSS: Primary Sjogren's Syndrome; sSS: Secondary Sjogren's Syndrome.

Table S1: Cytological analysis of salivary sediment in studied patients

Disease (number of patients)	Incidence rate, n (%)			
	Epithelial cells	Erythrocytes	Granulocytes	Lymphocytes

pSS[1](n=12)	11 (91.6%)	1 (8.3%)	5 (41.6%)	11 (91.6%)
sSS+RA[2] (n=13)	13 (100%)	1 (7.6%)	6 (46.2%)	10 (76.9%)
sSS+SLE[3] (n=8)	8 (100%)	1 (12.5%)	2 (25%)	6 (75%)
sSS+ SSD [4] (n=7)	7 (100%)	0 (0%)	2 (28.5%)	5 (71.4%)
Average for group	39 (97.5%)	3 (7.5%)	15 (37.5%)	32 (80%)
Control group (n=20)	11 (55%)	0 (0%)	1 (5%)	0 (0%)

[1]primary Sjogren's Syndrome
[2]secondary Sjogren's Syndrome, associated with rheumatoid arthritis
[3]secondary Sjogren's Syndrome, associated with systemic lupus erythematosus
[4]secondary Sjogren's Syndrome, associated with systemic scleroderma

Table S2: Lymphocytes percentage in the cellular composition of salivary sediment in studied patients

**Disease (number of patients)**	**Lymphocytes percentage in the cellular composition**			
	**0**	**1-10%**	**11-50%**	**>50%**
pSS[1](n=12)	1	7	3	1
sSS+RA[2] (n=13)	3	8	1	1
sSS+SLE[3] (n=8)	2	5	1	0
sSS+ SSD [4] (n=7)	2	3	2	0
Average for group	8	23	7	2

[1]primary Sjogren's Syndrome
[2]secondary Sjogren's Syndrome, associated with rheumatoid arthritis
[3]secondary Sjogren's Syndrome, associated with systemic lupus erythematosus
[4]secondary Sjogren's Syndrome, associated with systemic scleroderma

Table S3: Immunogram of parotid saliva of the studied patients.

**Disease (number of patients)**	**Incidence rate, %**				
	**T-cells (CD3+)**	**T-helpers (CD4+)**	**T-cytotoxic cells (CD8+)**	**B-cells (CD19+)**	**Natural killer cells (CD56+)**
pSS[1](*n* = 12)	90%	43.7%	64%	9%	9%
sSS+RA[2] (*n* = 13)	65%	45%	47.8%	2.2%	8.7%
sSS+SLE[3] (*n* = 8)	66.8%	36.4%	51.4%	0	0
sSS+SSD [4] (*n* = 7)	64.5%	41.5%	48.9%	0	0
Average for group	71.7%	41.6%	53%	2.8%	4.4%
Control group	0	0	0	0	0

[1] primary Sjogren's Syndrome; [2] secondary Sjogren's Syndrome, associated with rheumatoid arthritis; [3] secondary Sjogren's Syndrome, associated with systemic lupus erythematosus; [4] secondary Sjogren's Syndrome, associated with systemic scleroderma.
